# Highly efficient nuclear DNA typing of the World War II skeletal remains using three new autosomal short tandem repeat amplification kits with the extended European Standard Set of loci

**DOI:** 10.3325/cmj.2012.53.17

**Published:** 2012-02

**Authors:** Irena Zupanič Pajnič, Barbara Gornjak Pogorelc, Jože Balažic, Tomaž Zupanc, Borut Štefanič

**Affiliations:** Institute of Forensic Medicine, Faculty of Medicine, University of Ljubljana

## Abstract

**Aim:**

To perform an efficiency study of three new amplification kits with the extended European Standard Set (ESS) of loci for autosomal short tandem repeat (STR) typing of skeletal remains excavated from the World War II mass graves in Slovenia.

**Methods:**

In the beginning of the 2011, we analyzed 102 bones and teeth using the PowerPlex ESX 17 System (Promega), AmpFiSTR NGM PCR Amplification Kit (Applied Biosystems), and Investigator ESSplex Kit (Qiagen). We cleaned the bones and teeth, removed surface contamination, and ground them into a powder using liquid nitrogen. Prior to DNA isolation with Biorobot EZ1 (Qiagen), 0.5 g bone or tooth powder was decalcified. Nuclear DNA of the samples was quantified using real-time polymerase chain reaction. All three kits used the same extract with the amplification conditions recommended by the manufacturers.

**Results:**

We extracted up to 131 ng DNA/g of powder from the bones and teeth. All three amplification kits showed very similar efficiency, since DNA typing was successful with all amplification kits in 101 out of 102 bones and teeth, which represents a 99% success rate.

**Conclusion:**

The commercially available ESX 17, ESSplex, and NGM kits are highly reliable for STR typing of World War II skeletal remains with the DNA extraction method optimized in our laboratory.

DNA typing of bone and tooth samples has been successfully used in anthropological studies and forensic identification analysis (1,2). Nuclear DNA is the preferred genome of amplification for forensic purposes as it is individually specific and provides bi-parental kinship information (3). The success of DNA typing in old bones and teeth is often limited by small amounts of endogenous DNA, presence of polymerase chain reaction (PCR) inhibitors, DNA degradation, and an exceptional risk of contamination (4-6). Mitochondrial DNA testing has been regularly employed in the forensic identification of aged skeletal remains (7-10). Recently, some articles have reported a successful typing of nuclear short tandem repeats (STR) from ancient material using an increased number of cycles (11-18). In 2009 and 2010, new amplification kits were developed to meet the European Network of Forensic Institutes and the European DNA Profiling group recommendations for increasing the European Standard Set (ESS) of loci to improve its discrimination power and to fulfill the increasing requirements regarding sensitivity and reproducibility for the analysis of minute amounts of DNA by adopting five additional mini-STRs: D2S441, D10S1248, D22S1045, D1S1656, and D12S391 (19,20). Some validation, concordance, and population studies (21-28) have been published for new amplification kits with the extended ESS of loci. It was shown that the new kits are robust enough to genotype degraded DNA samples through the use of mini STR loci and have increased tolerance to common inhibitors and increased sensitivity to obtain full profiles from low-level DNA samples from casework (27,29,30). However, no study has been performed using new amplification kits on old skeletal remains. We attempted to obtain autosomal STR profiles from the World War II bones and teeth with three new commercially available amplification kits with the extended ESS of loci using the PCR protocols recommended by the manufacturers without increasing the number of cycles or any other modification of protocols.

## Materials and methods

This study analyzed 102 bones and teeth excavated from five World War II mass graves in Slovenia. The Commission on Concealed Mass Graves in Slovenia has recently registered almost 600 hidden mass graves from that period (31). There is no precise data on the number of Yugoslav communist armed forces victims in Slovenia but the number of missing persons could be as high as 100 000. We analyzed the bones and teeth from the Konfin I (13), Konfin II, Storžič (14), Bodoveljska Grapa, and Mozelj mass graves. From Konfin I mass grave, we analyzed 57 femurs and 12 tibias and from Konfin II 17 teeth (12 molars and 5 premolars). From Storžič grave, we analyzed 3 femurs and 1 molar, from Bodoveljska Grapa 10 femurs, and from Mozelj grave 2 femurs. A total of 84 bone samples (72 femurs and 12 tibias) and 18 tooth samples were evaluated.

We performed a comparative analysis of DNA preservation in skeletal remains from different mass graves according to the results of quantification. Since femurs were typed for all mass graves except Konfin II, the comparison of DNA preservation in femurs was made with four mass graves.

We followed the published recommendations to ensure the quality standards and to prevent contamination in the molecular genetics laboratory (17,32-37). In the case of ancient DNA, there are several main sources of contamination, including excavators and anthropologists who handle the remains, airborne contaminants from the laboratory, and contaminants present in laboratory reagents or on consumable items (38). Therefore, we created an elimination database of STR genetic profiles for each mass grave that allowed traceability in the event of contamination. In the databases, we included everyone who had been in contact with the skeletal remains in any phase of the working process (excavation, storage, anthropological analysis, or molecular genetic analysis). We also included extraction-negative controls in every batch of extraction (usually 23 samples) and PCR-negative controls in every amplification reaction to verify the purity of the extraction and amplification reagents and plastics. In five batches of extraction, five extraction-negative controls were processed.

### DNA extraction

We collected buccal smears on sterile cotton swabs from persons included in the elimination databases. The bone and tooth samples for DNA analysis were collected, labeled, and photo-documented. For genetic investigations, a 5- to 10-cm fragment was taken from each femur and tibia. Thirteen molars and 5 premolars were removed from 6 upper and 8 lower jawbones.

Bone samples were cleaned mechanically and chemically, while tooth samples were cleaned chemically and irradiated with UV light for 2 × 30-minute with the tooth rotated 180° between each exposure prior to grinding into a powder. The bone surface was decontaminated by the physical removal of the surface using a rotary sanding tool (Dremel, Breda, the Netherlands) and liquid nitrogen. The bones and teeth were rinsed in 5% Alconox detergent (Sigma-Aldrich, St. Louis, MO, USA), water, and 80% ethanol. Grinding followed in a TissueLyser (Retsch, Haan, Germany) homogenizer using liquid nitrogen. The whole procedure was carried out in a room designed exclusively for processing old skeletal remains. Mechanical cleaning was performed in a closed citostatic C-(MaxPro)^3^-130 (Iskra Pio) safety cabinet.

Genomic DNA was obtained from 0.5 g of bone or tooth powder according to Zupanič Pajnič et al (13,39). After 72 hours of decalcification, we usually obtained a precipitate with incompletely decalcified bone powder. DNA was purified in a Biorobot EZ1 device (Qiagen, Hilden, Germany) using the EZ1 DNA Investigator Card and the EZ1 DNA Investigator Kit (Qiagen). The Biorobot EZ1 was used to obtain genomic DNA from decalcified bone and tooth precipitate using the large-volume protocol following the manufacturer’s instructions (40), and from the elimination database buccal swab samples using the “tip dance” protocol. The extraction-negative controls were included in the extraction process to verify the purity of the extraction reagents and plastics. The final volume of bone and tooth extracts was 50 µL, of which 2 µL was used for quantification, 17.5 µL for amplification of the STRs with the PowerPlex^®^ ESX 17 System (Promega, Madison, WI, USA), 10 µL for amplification with the AmpFlSTR^®^ NGM^TM^ PCR Amplification Kit (Applied Biosystems, Foster City, CA, USA), and 17.1 µL for the amplification with the Investigator ESSplex Kit (Qiagen).

### DNA quantification

The DNA extracts from all the bone and tooth samples were quantified using the Quantifiler^TM^ Human DNA Quantification Kit (Applied Biosystems). The reactions were carried out in an ABI PRISM 7000 Sequence Detection System (Applied Biosystems) using the SDS software, version 1.0 (Applied Biosystems) according to the manufacturer’s instructions (41).

### Autosomal STR typing

STR typing of the autosomal DNA was performed for the bones and teeth using the three amplification kits. All these kits amplify 15 polymorphic STR markers (D1S1656, D2S441, D2S1338, D3S1358, D8S1179, D10S1248, D12S391, D16S539, D18S51, D19S433, D21S11, D22S1045, FGA, TH01, vWA) and sex-specific amelogenin simultaneously in a single PCR. The ESX 17 also contains the SE33 locus. All three multiplex kits analyzed the same extract. The amplification protocols and thermal cycling conditions were in accordance with the manufacturer’s instructions (42-44) using the ABI PRISM 7000 Sequence Detection System (Applied Biosystems). For the ESX 17, PCR reactions were performed with at most 17.5 µL DNA, and 1 ng of DNA was amplified for the samples with a concentration <60 pg/µL. For the NGM kit, PCR reactions were performed with at most 10 µL DNA, and 1 ng of DNA was amplified for the samples with a concentration <100 pg/µL. For the ESSplex kit, PCR reactions were performed with at most 17.1 µL DNA, and 1 ng of DNA was amplified for the samples with a concentration <60 pg/µL. Simultaneously with the forensic samples, we amplified the positive control (Control DNA 9947A for the ESX 17 and NGM kit, and Control DNA XY13 for the ESSplex kit) and negative PCR controls, as well as the extraction negative controls where the maximum volume of extracts was used for amplification. The fluorescent-labeled products of the amplification kits were separated using 10 seconds injection time and 3 kV injection voltage on an automatic ABI PRISM^TM^ 3130 Genetic Analyzer (Applied Biosystems) using the 3130 Performance Optimized Polymer 4 (Applied Biosystems) and the GeneScan-500 LIZ (Applied Biosystems) internal size standard with the NGM kit, CC5 Internal Lane Standard 500 (Promega) with the ESX 17, and DNA size standard 550 BTO (Qiagen) with the ESSplex kit. The genetic profiles were determined using the Data Collection, version 3.0 and GeneMapper ID version 3.2 (Applied Biosystems) computer software with a 50 relative fluorescence units peak amplitude threshold for all dyes.

STR typing was also carried out for persons who were included in the elimination databases using the Identifiler or NGM kit (Applied Biosystems). Their genetic profiles were compared with those obtained from the bones and teeth to monitor possible contamination of the bone and teeth samples with modern-day DNA.

## Results

We detected more than 23 pg DNA/µL of isolate from 66 bones and teeth (Table 1), less than 23 pg DNA/µL of isolate from 35 bones and teeth, and no DNA from one femur (Table 1). We extracted up to 94 ng DNA/g of powder from the teeth, up to 26.4 ng DNA/g of powder from the tibias, and up to 131 ng DNA/g of powder from the femurs (Table 1).

**Table 1:** Nuclear DNA quantity (the Quantifiler^TM^ Human DNA Quantification Kit), expressed in pictograms of DNA per microliter of isolate, and the efficiency of autosomal short tandem repeats (STR) typing (the PowerPlex^®^ ESX 17 System, the AmpF*l*STR^®^ NGM^TM^ PCR Amplification Kit, and the Investigator ESSplex Kit), expressed as the number of successfully typed autosomal STRs in 102 World War II bones and teeth

		Efficiency of STR typing by		
Tooth/bone sample	Quantity (pg/µL)	ESX 17	NGM	ESSplex
tooth 1*	28	17/17	16/16	16/16
tooth 2*	57	17/17	16/16	16/16
tooth 3*	79	17/17	16/16	16/16
tooth 4*	272	17/17	16/16	16/16
tooth 5*	127	17/17	16/16	16/16
tooth 6*	116	17/17	16/16	16/16
tooth 7*	104	17/17	16/16	16/16
tooth 8*	65	17/17	16/16	16/16
tooth 9*	below 23	16/17	14/16	16/16
tooth 10*	170	17/17	16/16	16/16
tooth 11*	363	17/17	16/16	16/16
tooth 12*	75	17/17	16/16	16/16
tooth 13*	below 23	17/17	16/16	16/16
tooth 14*	294	17/17	16/16	16/16
tooth 15*	143	17/17	16/16	16/16
tooth 16*	107	17/17	16/16	16/16
tooth 17*	150	17/17	16/16	16/16
tooth 18^†^	940	17/17	16/16	16/16
tibia 1^‡^	below 23	15/17	10/16	14/16
tibia 2^‡^	below 23	17/17	16/16	16/16
tibia 3^‡^	55	17/17	16/16	16/16
tibia 4^‡^	112	17/17	16/16	16/16
tibia 5^‡^	40	17/17	16/16	16/16
tibia 6^‡^	35	17/17	16/16	16/16
tibia 7^‡^	below 23	14/17	9/16	14/16
tibia 8^‡^	below 23	14/17	11/16	12/16
tibia 9^‡^	34	17/17	16/16	15/16
tibia 10^‡^	264	17/17	16/16	16/16
tibia 11^‡^	24	17/17	16/16	16/16
tibia 12^‡^	250	17/17	16/16	16/16
femur A ant^‡^	383	17/17	16/16	16/16
femur B ant^‡^	below 23	17/17	15/16	15/16
femur C ant^‡^	100	17/17	16/16	16/16
femur D ant^‡^	49	17/17	16/16	16/16
femur E ant^‡^	31	17/17	16/16	16/16
femur F ant^‡^	below 23	17/17	16/16	16/16
R femur 1^‡^	114	17/17	16/16	16/16
R femur 2^‡^	27	17/17	15/16	16/16
R femur 3^‡^	60	17/17	16/16	16/16
R femur 4^‡^	23	17/17	16/16	16/16
R femur 5^‡^	35	17/17	16/16	16/16
R femur 6^‡^	30	17/17	16/16	16/16
R femur 7^‡^	50	17/17	16/16	16/16
R femur 8^‡^	below 23	17/17	15/16	16/16
R femur 10^‡^	below 23	17/17	16/16	16/16
R femur 11^‡^	59	17/17	16/16	16/16
R femur 12^‡^	75	17/17	16/16	15/16
R femur 13^‡^	below 23	17/17	16/16	16/16
R femur 14^‡^	below 23	17/17	11/16	15/16
R femur 15^‡^	102	17/17	16/16	16/16
R femur 16^‡^	below 23	17/17	14/16	16/16
R femur 17^‡^	below 23	16/17	13/16	12/16
R femur 18^‡^	1310	17/17	16/16	16/16
R femur 19^‡^	288	17/17	16/16	16/16
R femur 21^‡^	84	17/17	16/16	16/16
R femur 22^‡^	48	17/17	16/16	16/16
R femur 23^‡^	57	17/17	16/16	16/16
R femur 24^‡^	159	17/17	16/16	16/16
R femur 25^‡^	below 23	17/17	16/16	16/16
R femur 26^‡^	24	17/17	12/16	16/16
R femur 27^‡^	93	17/17	16/16	16/16
R femur 28^‡^	below 23	17/17	16/16	16/16
R femur 29^‡^	below 23	17/17	16/16	15/16
R femur 30^‡^	below 23	17/17	16/16	16/16
R femur 32^‡^	76	17/17	16/16	16/16
R femur 34^‡^	52	17/17	16/16	16/16
R femur 38^‡^	38	17/17	16/16	16/16
R femur 42^‡^	below 23	16/17	14/16	15/16
R femur 43^‡^	below 23	17/17	14/16	16/16
R femur 45^‡^	below 23	16/17	16/16	15/16
R femur 47^‡^	33	17/17	16/16	16/16
R femur 48^‡^	below 23	17/17	16/16	16/16
R femur 49^‡^	94	17/17	16/16	16/16
R femur 50^‡^	24	17/17	16/16	16/16
R femur 51^‡^	53	17/17	16/16	16/16
R femur 53^‡^	below 23	17/17	16/16	16/16
R femur 55^‡^	77	17/17	16/16	16/16
R femur 57^‡^	57	17/17	16/16	16/16
R femur 58^‡^	29	16/17	15/16	15/16
R femur 59^‡^	33	17/17	16/16	16/16
R femur 60^‡^	32	17/17	16/16	16/16
R femur 61^‡^	below 23	17/17	16/16	16/16
R femur 63^‡^	27	17/17	16/16	16/16
R femur 65^‡^	undetected	0/17	0/16	0/16
R femur 67^‡^	46	17/17	16/16	16/16
R femur 68^‡^	below 23	15/17	14/16	13/16
R femur 69^‡^	32	17/17	16/16	16/16
L femur 1^†^	below 23	17/17	16/16	16/16
L femur 2^†^	34	17/17	16/16	16/16
L femur 3^†^	below 23	17/17	16/16	16/16
L femur 1^§^	42	17/17	16/16	16/16
L femur 4^§^	below 23	16/17	8/16	13/16
L femur 5^§^	below 23	17/17	15/16	16/16
L femur 7^§^	below 23	17/17	14/16	16/16
L femur 9^§^	below 23	14/17	7/16	12/16
L femur 11^§^	36	17/17	16/16	16/16
L femur 14^§^	40	17/17	16/16	16/16
L femur 15^§^	below 23	17/17	16/16	16/16
L femur 16^§^	below 23	17/17	16/16	16/16
L femur 20^§^	below 23	16/17	9/16	15/16
R femur 4^║^	below 23	17/17	16/16	16/16
R femur 5^║^	26	16/17	15/16	16/16

*Konfin II mass grave.

^†^Storžič mass grave.

‡Konfin I mass grave.

§Bodoveljska Grapa mass grave.

║Mozelj mass grave.

The typing of autosomal STR loci with the ESX 17, ESSplex, and NGM was successful in all but one sample. In this bone sample, no detectable DNA was observed and all three STR typing kits failed to obtain the result at any locus. In 101 successfully typed bone and teeth samples, we obtained full profiles in 86% of the samples with the ESX 17 (with amplification product at all STR loci and amelogenin), 83% of the samples with the ESSplex, and 78% of the samples with the NGM kit. We obtained partial profiles for 13 out of the 102 bones and teeth using the ESX 17, 16 bones and teeth using the ESSplex, and 21 bones and teeth using the NGM kit (Table 1).

Full ESX 17-STR genetic profiles were obtained from 62 femurs, 9 tibias, and 17 teeth (Table 1). In 13 bones and teeth with partial profiles, the loci that were not amplified were primarily the longest loci D18S51, D16S539, FGA, or SE33. Full ESSplex-STR genetic profiles were obtained from 59 femurs, 8 tibias, and all 18 teeth (Table 1). In 16 bones with partial profiles, the loci that were not amplified were primarily the longest loci D21S11, D2S1338, FGA, and D8S1179. Full NGM-STR profiles were obtained from 54 femurs, 9 tibias, and 17 teeth (Table 1). In 21 bones and teeth with partial profiles, the loci that were not amplified were primarily the longest loci D2S1338, D18S51, and FGA.

Since we minimized the possibility of contamination during genetic investigations, very low levels of exogenous DNA contamination were observed in some extraction-negative controls and no contamination was noted in the PCR-negative controls for all three ESS STR amplification kits. The authenticity of the genetic profiles of the bones and teeth was confirmed by their mismatch with persons from the elimination databases and the identical genetic profiles obtained using the ESX 17, NGM, and ESSplex kit.

Femurs were less preserved in the Bodoveljska Grapa, Mozelj, and Storžič grave and better preserved in the karst cave Konfin I (Table 1). More full profiles were obtained from teeth than from bones (up to 100% with ESSplex kit and up to 75% with ESX 17 and NGM). Among the long bones, more full profiles were obtained from femurs than tibias (up to 86% with ESX 17 and up to75% with ESX 17 and NGM). We obtained full profiles for all the teeth with ESSplex kit and for 94% of the teeth with ESX 17 and NGM kits. We obtained full profiles for 86% of the femurs using ESX 17, 82% using ESSplex kit, and 75% using NGM kit. We obtained full profiles for 75% of the tibias with the ESX 17 and NGM kit, and 67% with the ESSplex kit (Table 1).

## Discussion

The efficiency study indicated that commercially available ESX 17, ESSplex, and NGM kits were highly reliable for STR typing of World War II skeletal remains, since all three kits very often produced complete STR profiles of autosomal DNA. Typing of low-level DNA samples from casework with new ESS amplification kits also showed better results in comparison with older amplification kits (27,29,30).

The skeletons uncovered from Slovenian mass graves had undergone various levels of environmental insult due to the different environments they were exposed to, indicating that the environment seems to be an important factor of long-term DNA survival. Some of the bodies were buried in soil and some of them were thrown into karst caves. The temperature, humidity, pH and geochemical properties of the soil, and the presence of microorganisms affect the preservation of the DNA in skeletal remains (45). One of the key parameter of long-term DNA survival is the thermal history of the sample and it was early recognized that a favorable factor are low mean annual temperatures (46). Accordingly, skeletal remains were best preserved in the karst caves. Teeth and femurs had better preserved DNA and showed better STR typing results than tibias. These findings are in concordance with those reported by Miloš et al (47), Misner et al (48), and Edson et al (49).

Advanced extraction and purification techniques were found to be essential tools for obtaining sufficient DNA from bones and teeth uncovered from Slovenian World War II mass graves. We extracted up to 131 ng DNA/g of powder from the WWII bones and teeth. Extraction and purification methods using the EZ1 Biorobot, together with more sensitive and robust new amplification kits with the ESS loci that are more tolerant to common inhibitors, allowed us to overcome the challenges associated with processing compromised skeletal remains and ultimately obtain STR DNA profiles in 99% of the bones and teeth. Only for one bone STR typing with new amplification kits failed. When dealing with old skeletal remains, all three kits can be used very successfully without any changes to the manufacturers’ PCR amplification protocols.
